# Supplementary material for: Cryo-EM structure and biochemical characterization of a BRAF/CRAF heterodimer: Negative charge in the NtA motif is not required for RAF activation
Source: bioRxiv. 2026 May 14:2026.05.11.724350. Preprint. [Version 1] doi: 10.64898/2026.05.11.724350 (PMC13192968; doi:10.64898/2026.05.11.724350)
Supplement: Supplement 1 [file media-1.pdf]

Supplementary Materials for

Cryo-EM structure and biochemical characterization of a BRAF/CRAF heterodimer: Negative charge in the NtA motif is not required for RAF activation

Byung Hak Ha[1,2], Emre Tkacik[1,2], Dimitrios Gazgalis[1,2], Hyelim Kang[1], Dong Man Jang[1,2], Sayan Chakraborty[1,2], , Hyesung Jeon[1,2], and Michael J. Eck[1,2†]

[1]Department of Cancer Biology, Dana-Farber Cancer Institute, Boston, MA 02215, USA.
[2]Department of Biological Chemistry and Molecular Pharmacology, Harvard Medical School, Boston, MA 02115, USA.

[†]Corresponding Author:
Michael J. Eck
e-mail: Michael_Eck@dfci.harvard.edu

This file includes:

Supplemental Table 1

Supplemental Figures 1 to 5

Supplementary Table 1. Cryo-EM data collection, refinement and validation statistics

**Data collection and processing**		
Magnification	105,000x	
Voltage (kV)	300	
Electron exposure (e/Å^2)	62.5	
Defocus range (μm)	- 0.8 to -2.2	
Pixel size	0.825	
Symmetry imposed	C1	
Number of micrographs	9,676	
**Refinement**	BRAF/CRAF^{FERA}/14-3-3 complex	BRAF/CRAF^{FERA}/MEK1^{SASA}/14-3-3 complex
PDB	13DU	13DV
EMD	EMD-77011	EMD-77012
Final particle images (no.)	139,194	171,208
Map resolution (Å)		
0.143 FSC threshold	3.3	3.9
Initial model used	6NYB	6NYB, 6PP9
Model composition		
Chains	4	5
Non-hydrogen atoms	8,301	10,399
Protein residues	1,028	1,295
Ligands	29L (2)	29L (2), LCJ (1)
B factors (Å^2)		
Proteins	65.5	112.0
Ligands	45.2	97.6
R.m.s deviations		
Bond length (Å)	0.002	0.003
Bond angles (°)	0.457	0.570
Validation		
MolProbity score	2.83	3.41
Ramachandran plot		
Favored (%)	94.85	90.81
Outliers (%)	0	0
Model vs. Data		
CC (mask)	0.76	0.64
CC (box)	0.71	0.68
CC (peaks)	0.64	0.52
CC (volume)	0.75	0.64
Mean CC for ligands	0.76	0.63

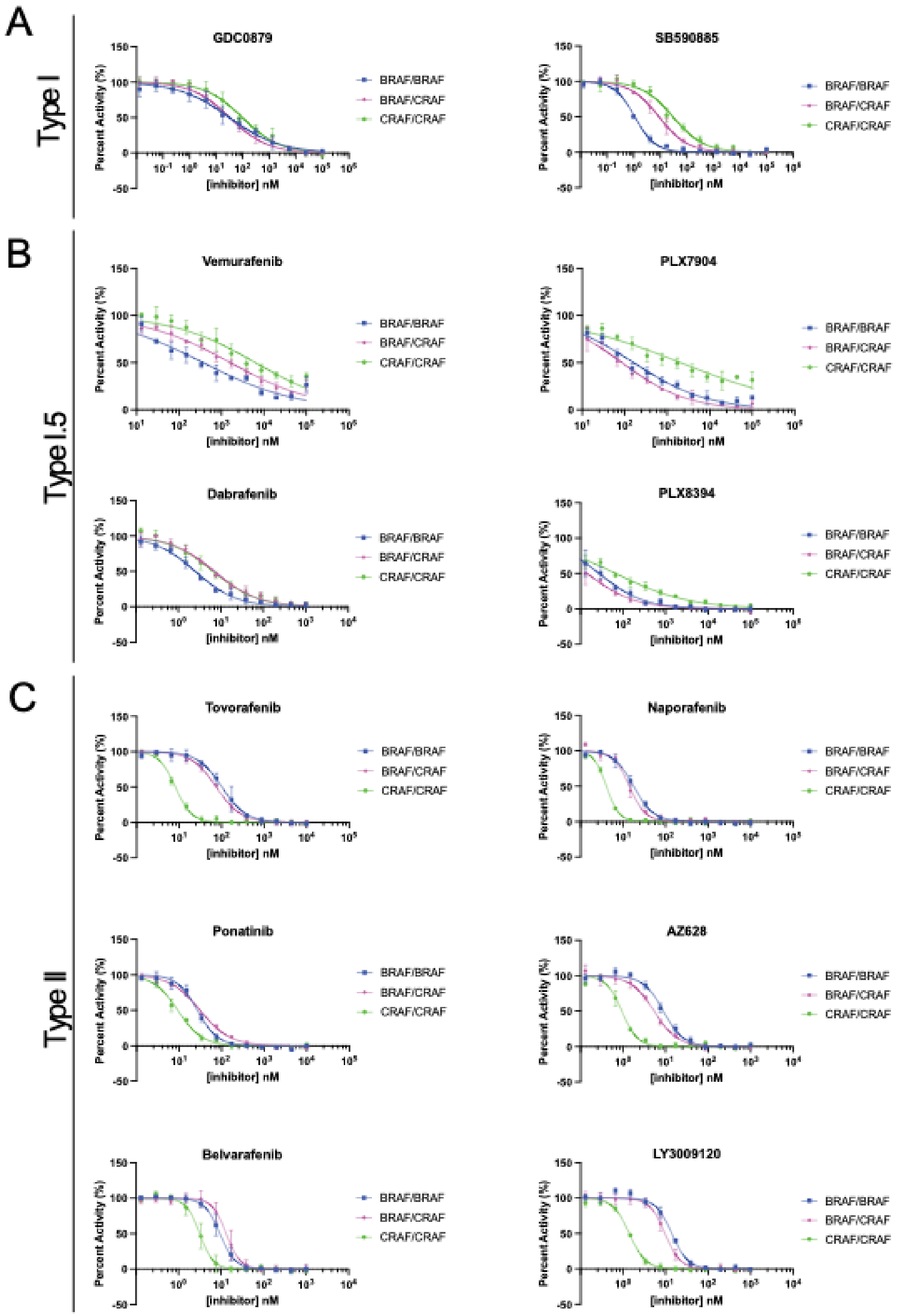

Supplementary Figure 1. Profiling of RAF inhibitors across BRAF and CRAF homodimers and heterodimers. Representative concentration-response curves used to determine IC_{50} values provided in Table 1 of the main text. Data are plotted as mean ± standard deviation from one independent experiment performed in triplicate (n≥3).

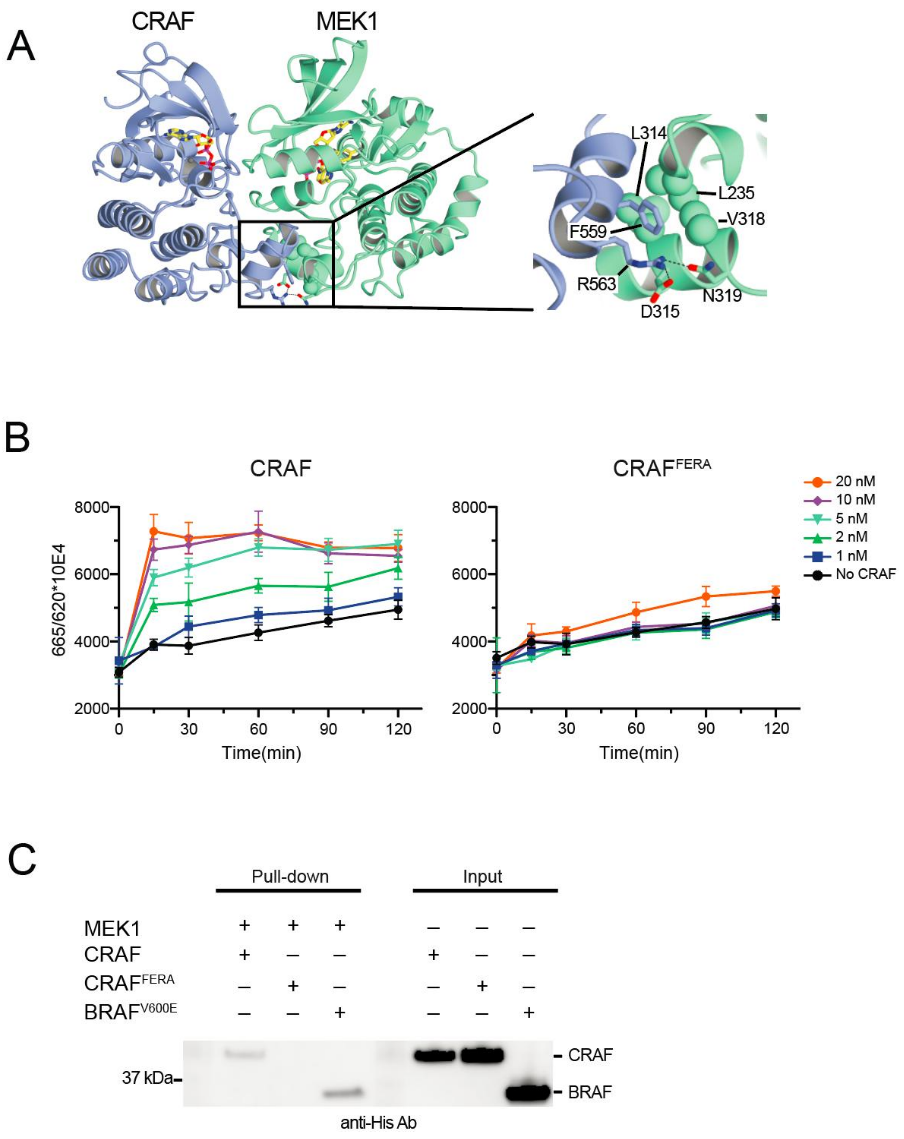

Supplementary Figure 2. Design and evaluation of a CRAF variant that does not bind MEK. A, Guided by our prior cryo-EM structure of CRAF and MEK1 (PDB: 9MMQ), we introduced the F559E/R563A double mutation in the αG-helix of CRAF to prevent its association with MEK1. We refer to the resulting construct as CRAF^{FERA} and it also contains the SSDD substitution of the NtA motif. CRAF is shown in light blue and MEK1 in green, and the MEK1 residues interacting with F559 and R563 are indicated. B, The activity of CRAF and CRAF^{FERA} was measured in a TR-FRET assay, varying the concentration of CRAF and the incubation time. As expected, CRAF^{FERA} was markedly impaired in its ability to phosphorylate MEK1. Values plotted are mean ± standard deviation (n=3) C, Pull-down assay testing binding of CRAF, CRAF^{FERA}, or BRAF^{V600E} to bead-immobilized MEK1. MEK1 with an N-terminal strep tag was first immobilized on strep beads at 4°C. The MEK1-bound beads were then incubated with His$_6$-tagged CRAF, CRAF^{FERA}, or BRAF^{V600E} for 1 hour at 4°C. After three washing steps, the beads were eluted with SDS-PAGE sample buffer and bound proteins were analyzed by SDS-PAGE and blotting with an anti-His$_5$ antibody. The 37 kDa molecular weight marker is indicated. The three lanes on the right show 10% of the input of each RAF protein.

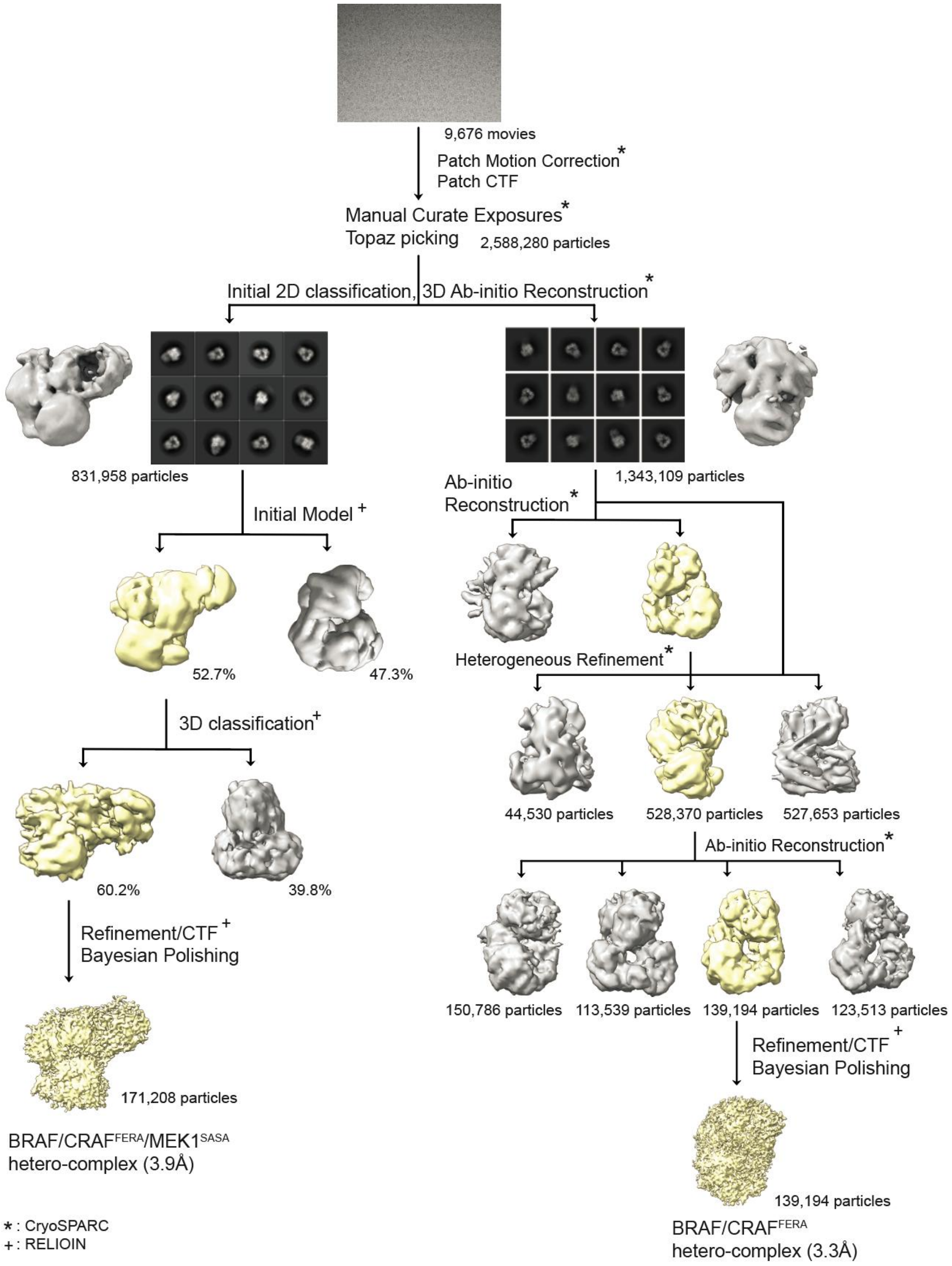

Supplementary Figure 3. Cryo-EM image processing workflow. A total of 9,676 movie micrographs were recorded using a Thermo Fisher Titan Krios cryo-TEM equipped with a K2 direct electron detector and Gatan Quantum Image filter. Patch motion correction and patch CTF correction were performed using CryoSPARC, followed by manual curation of the exposures and particle picking with Topaz. This resulted in the selection of 2,588,280 particles, which were then subjected to 2D classification and initial model generation using Ab-initio methods. The selected particles were transferred to RELION and processed through CTF refinement and Bayesian polishing to produce the final map. "*" indicates processes performed in CryoSPARC, and "+" indicates processes processed using RELION.

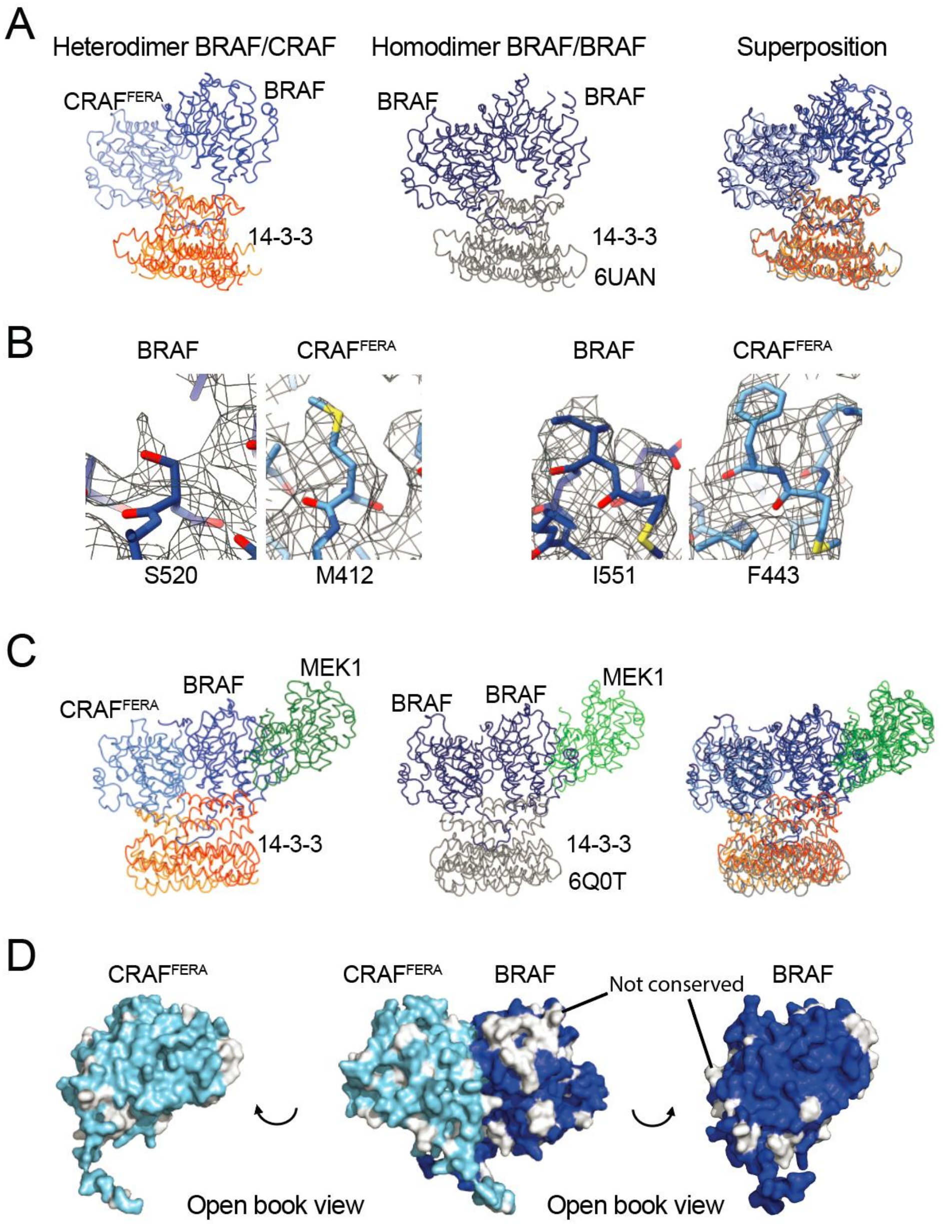

Supplementary Figure 4. Additional structural analysis of the BRAF/CRAF heterodimer. A, Comparison of the BRAF/CRAF/14-3-3 heterodimer structure with that of the previously determined BRAF homodimer (PDB: 6UAN). Superposition of the two cryo-EM structures is shown on the far right (r.m.s.d. = 1.65Å for 1007 aligned Cα atoms). B, Cryo-EM density for selected sidechains that differ in size in BRAF versus CRAF. Differences in sidechain density for these and other residues guided assignment of the corresponding protomers in the heterodimer. C, Comparison of the MEK-bound BRAF/CRAF/14-3-3 heterodimer structure with that of the previously determined BRAF/MEK1/14-3-3 homodimer structure with a single MEK1 bound (PDB: 6Q0T). Superposition of the two cryo-EM structures is shown on the far right (r.m.s.d. = 1.65Å for 1007 aligned Cα atoms). D, "Open-book" view of the BRAF/CRAF dimer interface. Residues that are not identically conserved between BRAF and CRAF are shown in white on the surface models of BRAF (blue) and CRAF (light blue). Note that the interacting surfaces revealed in the open book views on the far left (for CRAF) and far right (for BRAF) are entirely conserved.

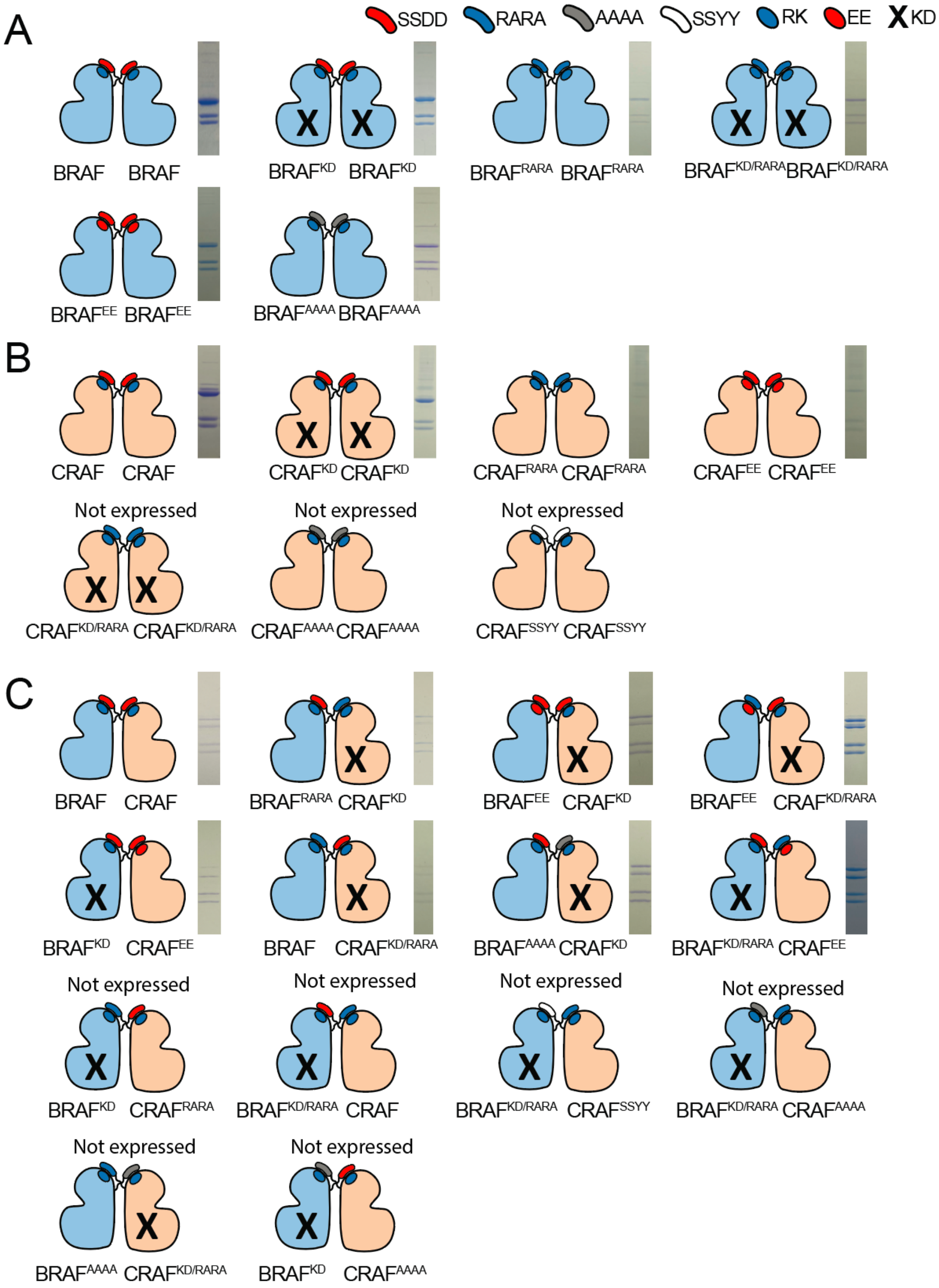

Supplementary Figure 5. Design of a structure-function experiment to test the role of the NtA/RKTR motif interaction in RAF homodimers and heterodimers. The key at the top of the figure identifies substitutions of the NtA and RKTR motifs in the context of (A) BRAF homodimers, (B) CRAF homodimers, or (C) BRAF/CRAF heterodimers. The NtA substitutions indicate the four-residue sequence of the NtA motif and the RKTR substitutions indicate the first two residues of this motif (RK or its replacement with EE). The "X" mark indicates a kinase-dead (KD) mutation (D576N in BRAF, D468N in CRAF). The corresponding lane of a Coomassie-stained SDS-PAGE gel is provided for the variants that were successfully expressed and purified as 14-3-3-bound dimers from insect cells. Activity assays for selected complexes are shown in Figure 3 in the main text.
